# A novel nano-formulation of olmesartan medoxomil with improved delivery and efficacy in the treatment of indomethacin-induced duodenitis in rats

**DOI:** 10.1590/1414-431X2023e12665

**Published:** 2023-05-29

**Authors:** H.A. Murad, T.M. Alqurashi

**Affiliations:** 1 Department of Pharmacology, Faculty of Medicine, Rabigh campus, King Abdulaziz University, Jeddah, Saudi Arabia; 2 Department of Pharmacology, Faculty of Medicine, Ain Shams University, Cairo, Egypt

**Keywords:** Indomethacin, Zeinmersomes, Olmesartan medoxomil, Duodenitis, Survivin

## Abstract

There are few studies addressing duodenal inflammation. This study was designed to investigate the effects of a recently developed biotechnological product, a nano-formulation of olmesartan medoxomil (OM) - olmesartan medoxomil zeinmersomes (OMZ) - for the treatment of indomethacin-induced duodenitis in rats. Adult male Wistar rats were given indomethacin (10 mg/kg/day) for four weeks. They were divided into a positive control group (PC, untreated) and two groups treated orally with 3 mg/kg per day of OM or OMZ for the last two weeks of the 4-week indomethacin-treatment. At end of the four weeks, blood and duodenum were collected. Duodenal homogenate was used for measurement of levels of myeloperoxidase, tumor necrosis factor-α (TNF-α), interleukin-6 (IL-6), malondialdehyde, reduced glutathione (GSH), and cleaved caspase-3. Duodenal sections were stained with H&E. Gene expressions of nuclear factor kappa B (*NF-κB p65*), Bcl-2-associated X protein (*Bax*), and B-cell lymphoma 2 (*Bcl-2*) by RT-PCR, and protein expression of survivin by western blot were assessed. Plasma and duodenal olmesartan concentrations were measured by high performance liquid chromatography mass spectrometry. The duodenitis rats showed significantly higher duodenal levels of myeloperoxidase, TNF-α, IL-6, malondialdehyde, and cleaved caspase-3, a significantly lower GSH level, and histopathological alterations. Moreover, they showed upregulated gene expressions of *NF-κB p65* and *Bax*, downregulated gene expression of *Bcl-2*, decreased *Bcl-2*/*Bax* ratio, and lower protein expression of survivin. OMZ was more effective in protecting the duodenum from indomethacin-induced injuries compared to OM due to improved delivery, higher bioavailability, and better anti-inflammatory, antioxidant, and antiapoptotic effects. OMZ could be a better choice for hypertensive patients with non-steroidal anti-inflammatory drugs-induced duodenitis.

## Introduction

Olmesartan medoxomil (OM) is an angiotensin receptor blocker (ARB) that has antihypertensive, anti-inflammatory, and other cardiovascular beneficial effects, but unfortunately it has low oral bioavailability due to its impaired water solubility and gastrointestinal (GI) efflux mechanisms. Recently, a novel biotechnological product of OM (OM-zeinmersomes, OMZ) was developed for treatment of liver fibrosis and associated duodenal alterations in rats, showing better bioavailability and better effectiveness compared with the regular form of OM (1,2). Duodenal inflammation (duodenitis) can result from prolonged use of non-steroidal anti-inflammatory drugs (NSAIDs), infections, Crohn's disease-associated complications, smoking, excessive alcohol intake, and other causes. It manifests as nonspecific inflammation like duodenal wall edema, leucocyte infiltration, luminal dilatation. If left untreated, this inflammation can become chronic or develop into duodenal ulceration (3). Indomethacin is an NSAID widely used to induce models of small intestine mucosal inflammation and ulceration. In rats, oral indomethacin induced diffuse jejuno-ileal mucosal inflammation and spot ulceration. The predominant mechanism of indomethacin-induced gut ulceration appears to be acute inflammation rather than ischemia (4).

Survivin, an antiapoptotic protein crucial for cell survival, is expressed in human and rat gastric mucosa. It is located in the surface epithelium that is exposed to harmful agents like NSAIDs, and in mucosal neck cells, which are progenitor cells, but has minimal expression in other cells of the gastric mucosa (5). In normal adult rats, survivin is strongly expressed in the epithelial cells of the small intestine, mainly in the crypt cells and marginally in the villi, and in the ascending colonic tissue. It is expressed in cells with a high proliferation rate and a remarkable capacity for regeneration (6). Apoptosis participates in the pathogenesis of NSAIDs-induced GI erosions and ulcers. Survivin protects against NSAIDs-induced GI mucosal injury (7). NSAIDs injure the gastric mucosa via suppression of prostaglandin production, downregulation of survivin, and increasing segregation of bile acids from the phosphatidylcholine lipids (8). Indomethacin dose-dependently reduced survivin expression in gastric mucosal cells and caused severe injury (9).

Studies addressing duodenal inflammation and ulceration are relatively few compared to other GI parts. Thus, this study was designed to detect effects of this novel nano-formulation (OMZ) in the treatment of indomethacin-induced duodenal inflammation in rats, which simulates NSAIDs-induced inflammation in the human gut.

## Material and Methods

### Olmesartan medoxomil zeinmersomes (OMZ) nano-formulation

The formulation, characterization, and *in vitro* diffusion studies of OMZ were performed as previously reported (2). Briefly, OM (0.1% w/v) and phospholipon 90G (0.5% w/v) were dissolved in ethanol. Zein (0.1% w/v) and polyethylene glycol-poly lactic acid-co-glycolic acid copolymer (0.2% w/v) were also dissolved in ethanol. Then, both solutions were blended and the ethanolic solution was evaporated. The deposited layer was kept in a vacuum cabinet overnight and deionized water was added to it. The dispersion was rotated at 100 rpm for 1 h at 45°C and then left aside for 1 h at 45°C to allow swelling of the vesicles. The resulting colloidal dispersion was ultrasonicated in an ice bath for 2 cycles of 8 min/cycle and the produced colloidal dispersion was lyophilized and stored under nitrogen. The OM encapsulation efficiency was determined. Ethanol was utilized for the solubilization of the sample and then passed through a 0.22-µm filter before being subjected to analysis protocol using high performance liquid chromatography (HPLC). The prepared OMZ was analyzed using Malvern ZSP (Malvern Panalytical, UK) and the formulations were diluted 20 times. The average particle size and zeta potential were assessed using three replicate samples. The prepared OMZ formula was investigated using a transmission electron microscope. The OMZ formula was subjected to *in vitro* diffusion studies using a 0.1-µm nylon diffusion membrane.

### Induction of indomethacin-induced duodenitis and experimental design

The study was approved by the Research Ethics Committee, Faculty of Pharmacy, King Abdulaziz University (PH-1443-63) and adhered to the international guidance for use of the laboratory animals. Adult male Wistar rats (200-250 g) were housed in cages for seven days before starting the procedures under environmental conditions at 24±1°C, about 50% humidity, 12-h light/dark cycle, and unrestricted access to standard food and water. The normal control (NC) group received phosphate buffered saline (PBS) as a vehicle, while three groups of rats (n=8) were given freshly prepared indomethacin (dissolved in PBS, 10 mg/kg per day orally) for four weeks about one hour before feeding (10). The three indomethacin groups included a positive control group (PC, untreated), OM group, which received OM suspended in distilled water with 0.25% w/v carboxymethyl cellulose (3 mg/kg per day), and OMZ group, which received the same dose of OMZ. Based on previous literature, the effect of treatments can be detected within two weeks; therefore, OM and OMZ were given orally in the last two weeks of the 4-week indomethacin-treatment duration. In a recently published paper (11), mice in the indomethacin group were given PBS orally for the first 7 days and then indomethacin for the last 5 days while mice in the treatment group were given chlorogenic acid for the whole period of the experiment (12 days) and indomethacin for the last 5 days. The H&E staining of the colon and ultrastructural examination of colonic microvilli have shown that chlorogenic acid protected against indomethacin-induced inflammation and mucosal damage. In another study (12), rats received olmesartan orally for a total of 9 days, given for 3 days before the induction of colitis by acetic acid and continued for 6 days after, including the induction day. The H&E staining showed that olmesartan ameliorated the acetic acid-induced ulcerative colitis. In the current study, indomethacin was given for 4 weeks in the positive control and treated groups while administration of OM and OMZ was started after the second week in the treated groups to give a chance for full development of the model because this study aimed to investigate the ability of treatments to reverse duodenitis. An early start of a treatment could just interfere with the development of the model rather than improve the condition after full establishment. At end of the four weeks, blood was collected and plasma was separated and kept at -80°C until analysis. Later, the rats were euthanized by decapitation under ketamine anesthesia and the duodenum was collected. Duodenal sections were subjected to histopathological examination while other parts of the duodenum were homogenized for measurement of levels of myeloperoxidase (MPO), tumor necrosis factor (TNF)-α, IL-6, malondialdehyde (MDA), reduced glutathione (GSH), and cleaved caspase-3. The gene expression of *NF-κB p65*, *Bax*, and *Bcl-2*, and the protein expression of survivin by western blot were evaluated. The olmesartan concentrations in plasma and duodenal homogenate were assessed by high performance liquid chromatography mass spectrometry (HPLC/MS).

### Measurement of inflammatory, oxidative, and apoptotic markers in duodenal homogenate

A part of the duodenum was washed and homogenized in PBS (0.01 M, pH 7.4) using a TissueLyser II (Qiagen, Germany) at 4°C to yield a 10% homogenate that was centrifuged at 12,000 g and 4°C for 15 min and the supernatant was kept at -80°C until analysis. The levels of MPO, TNF-α, IL-6, MDA, GSH, and cleaved caspase-3 were measured by ELISA kits (MyBioSource, Inc., USA). The duodenal protein content was measured, and the levels of the markers were expressed per mg protein.

### Histopathological examination

The duodenum was excised rapidly, washed with cold isotonic saline, and cut longitudinally. Duodenal samples were fixed in 10% phosphate-buffered formalin and embedded in paraffin. Paraffin blocks were put on ice, inserted into the microtome, and trimmed to expose the tissue surface. Duodenal sections (5 μm) were made, deparaffinized, rehydrated, mounted onto microscope slides, and stained with H&E. Three cuts (5 rats/group) were analyzed and the lesions were described as having mild, moderate, or severe damage as previously reported (13) (Table 1).

**Table 1:** Parameters and scores of histopathological findings.

Parameter	Score 1(Normal)	Score 2(Mild damage)	Score 3(Moderate damage)	Score 4(Severe damage)
Villus and crypt architecture	Normal villi and crypts	Marked preservation of villus and crypts architecture	Mild preservation of villus and crypts architecture	Marked deformity and hyaline degeneration of both villi and crypts
Surface epithelial integrity	Normal	Marked preservation	Mild preservation	Damaged with areas of epithelial loss
Goblet cells	Normal population	Moderate preservation	Mild preservation	Markedly lost
Inflammatory cell infiltration	Absent	Minimal	Moderate	Marked
Luminal mucous	Absent	Minimal amount	Moderate amount	Large amount

### Assessment of *NF-κB p65*, *Bax*, and *Bcl-2* gene expressions

Total RNA was isolated from the duodenum using a GeneJET kit (Thermo Fisher Scientific Inc., USA) and was reverse transcribed into cDNA using the RT-PCR kit (Invitrogen, USA) according to the manufacturer's instructions. Real time PCR was performed using an Applied Biosystems Cycler (USA) and SYBR green kit to assess the mRNA expression levels of *NF-κB p65*, *Bax*, and *Bcl-2*. The forward and reverse PCR primers used were as follows: NF-κB p65: 5′-CACCAAAGACCCACCTCACC-3′ and 5′-CCGCATTCAAGTATAGTCCC-3′; *Bax*: 5′-CCTGAGCTGACCTTGGAGCA-3′ and 5′-GGTGGTTGCCCTTTTCTACT-3′; *Bcl-2*: 5′-TGATAACCGGGAGATCGTGA-3′ and 5′-AAAGCACATCCAATAAAAAGC-3′, and *β-actin*: 5′-GTCAGGTCACTATCGGC-3′ and 5′-CATGGATGCCACAGGATTCC-3′. The real-time PCR conditions were followed as previously described (14-16). Data were normalized to *β-actin* and results are reported as fold change in relation to the negative control.

### Measurement of protein expression of survivin by western blot

Duodenal tissue was homogenized by incubation for 30 min in a lysis buffer, followed by centrifugation for 15 min at 12,000 *g* at 4°C. Total protein was extracted from the supernatant, and 100 μg/sample was separated on a gradient sodium dodecyl sulfate/polyacrylamide gel electrophoresis (SDS-PAGE) for assessing survivin expression and then proteins were transferred onto nitrocellulose membranes. The membrane was then cut into two pieces at a molecular weight of 30 kilodalton (kDa). The membrane part (<30 kDa) was incubated at 4°C overnight with a rabbit polyclonal anti-survivin antibody (Abcam, UK; ab469; dilution 1:500). The loading controls were performed by incubation of the other part with a rabbit polyclonal antibody to glyceraldehyde 3-phosphate dehydrogenase (GAPDH) (Abcam; ab9485; dilution 1:1000). Blots were incubated with secondary antibodies (Sigma Co., USA) for 60 min at room temperature (20°C). The survivin protein bands were developed by enhanced chemiluminescence reagent (Amersham Biosciences, USA), and bands were visualized by ChemiDoc Imaging System (Bio-Rad Laboratories Ltd., USA) (17,18).

### Determination of olmesartan concentration in plasma and duodenal homogenate

The levels of plasma and duodenal olmesartan were determined by HPLC/MS (19). The sample extraction and analysis were done as previously reported with slight modifications (20,21). Briefly, 200 μL of plasma or duodenal homogenate was mixed with 100 μL valsartan solution (100 ng/μL, used as an internal standard) and 700 μL acetonitrile. After centrifugation for 15 min at 12,000 *g* at 4°C, 200 μL of the supernatant was transferred to an autosampler vial, then 5.0 μL of the sample was injected for the liquid chromatography mass spectrometry coupled to diode array detection analysis. A calibration curve for olmesartan was done. Stock solutions for olmesartan and valsartan (1 mg/mL) were prepared in acetonitrile. A sequence of calibrator working solutions of olmesartan were made utilizing acetonitrile as the diluting agent. The concentrations of calibration solutions for olmesartan were 1.0-8000.0 ng/mL and for valsartan was fixed as 10 µg/mL. The peak area ratios of olmesartan to valsartan were found linear in the above-mentioned range of olmesartan concentrations.

### Statistical analysis

Data are reported as means±SD. Comparisons between groups were made using one-way analysis of variance (ANOVA) followed by Tukey test for multiple comparisons. The SPSS software (version 22, USA) was used to conduct the statistical analysis. The difference was considered significant when P<0.05.

## Results

### Formulation and characterization of OMZ nano-formula

The size analysis, zeta potential, and transmission electron microscopy of the OMZ formula are described in detail in our recent patent (2). Briefly, the prepared OMZ showed encapsulation efficiency of 95.73±3.28%. The average particle size was 137.8±6.4 nm, and the zeta potential was -17.5±3.61 mV. OMZ was spherical with a smooth surface. The *in vitro* diffusion pattern indicated the OM percentage that permeated from the prepared OMZ. The OMZ showed a controlled permeation of 98.21±4.46% over 48 h, while the regular OM permeated 97.5±13.07% over 4 h.

### Inflammatory and oxidative markers and cleaved caspase-3

In rats with duodenitis, the concentrations of MPO, TNF-α, IL-6, MDA, and cleaved caspase-3 significantly increased while the GSH level decreased in the duodenal homogenate. OMZ significantly improved these changes compared with OM (Table 2).

**Table 2:** Effects of olmesartan medoxomil raw drug (OM) and zeinmersomes formula (OMZ) on duodenal concentrations of MPO, THF-α, IL-6, MDA, GSH, and cleaved caspase-3 in indomethacin-induced duodenitis in rats (n=8).

	NC	PC	OM	OMZ
MPO (U/mg protein)	14.00±3.30	50.49±9.94^a^	28.64±4.32^a,b^	17.10±2.93^b,c^
TNF-α (pg/mg protein)	32.03±5.29	150.19±24.54^a^	72.33±9.80^a,b^	49.78±3.83^b,c^
IL-6 (pg/mg protein)	41.80±11.37	170.79±16.64^a^	76.09±11.37^a,b^	56.39±8.75^b,c^
MDA (nmol/mg protein)	50.49±9.94	221.72±25.67^a^	87.61±13.67^a,b^	63.38±9.68^b,c^
GSH (mmol/mg protein)	35.97±7.54	12.05±2.27^a^	21.70±4.61^a,b^	32.91±8.17^b,c^
Cleaved caspase-3 (ng/mg protein)	0.62±0.12	2.74±0.58^a^	1.65±0.48^a,b^	0.87±0.18^b,c^

Data are reported as means±SD. NC: Normal control, PC: Positive control. ^a^P<0.001 *vs* NC for all measurements. ^b^P<0.001 *vs* PC for all measurements except for GSH; ^b^P<0.05 (P = 0.019) OM *vs* PC. ^c^P<0.05 OMZ *vs* OM, equal to 0.003, 0.013, 0.018, 0.027, 0.005, and 0.002 for MPO, TNF-α, IL-6, MDA, GSH, and cleaved caspase-3, respectively (ANOVA).

### Gene expressions of *NF-κB p65*, *Bax*, and *Bcl-2*

**Figure 1:**
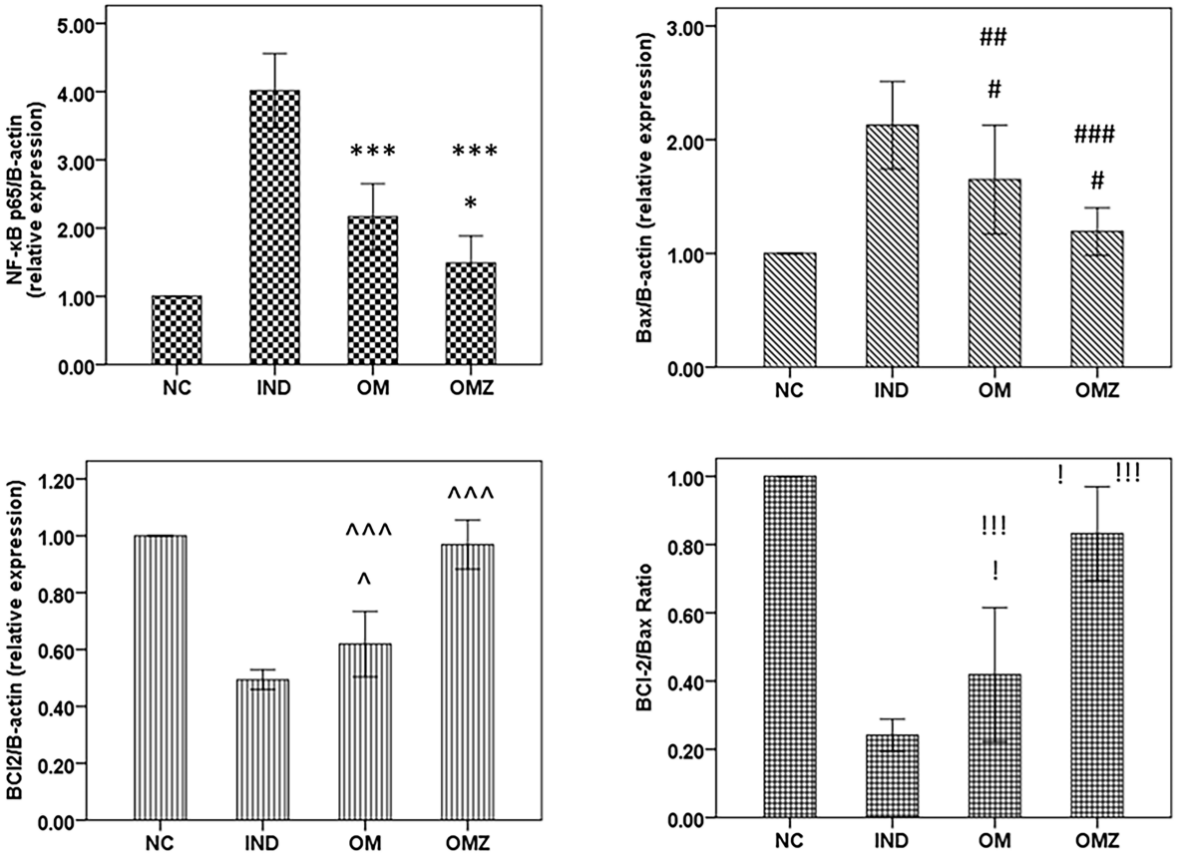
Effects of OM and OMZ on duodenal gene expressions of *NF-kB p65*, *Bax*, and *BCl-2* in indomethacin-induced duodenitis in rats (n=8). Data are reported as means±SD. NC: normal control; IND: indomethacin-induced duodenitis, untreated; OM: olmesartan medoxomil-treated group; OMZ: OM zeinmersomes-treated group. *P<0.05 OMZ *vs* OM; ***P<0.001 OM *vs* NC and OM & OMZ *vs* IND. ^#^P<0.05 OM *vs* IND & OMZ *vs* OM, ^##^P<0.001 OM *vs* NC, ^###^P<0.001 OMZ *vs* IND. ^ˆ^P<0.05 OM *vs* IND, ^ˆˆˆ^P<0.001 OM *vs* NC, OMZ *vs* OM & IND. ^!^P<0.05 OM *vs* IND, and OMZ *vs* NC, ^!!!^P<0.001 OM *vs* NC, OMZ *vs* IND, and OMZ *vs* OM (ANOVA).

In rats with duodenitis, the gene expressions of *NF-κB p65* and *Bax* increased while that of *Bcl*-2 gene and the *Bcl-2*/*Bax* ratio decreased in the duodenal homogenate. Both OM and OMZ significantly downregulated gene expressions of *NF-κB p65* and *Bax*, upregulated expression of *Bcl-2* gene, and increased the *Bax*/*Bcl-2* ratio. OMZ showed non-significant differences from NC except for the *B*ax/*Bcl-2* ratio where a slight but significant difference was detected (P=0.049) (Figure 1).

### Histopathological findings

**Figure 2:**
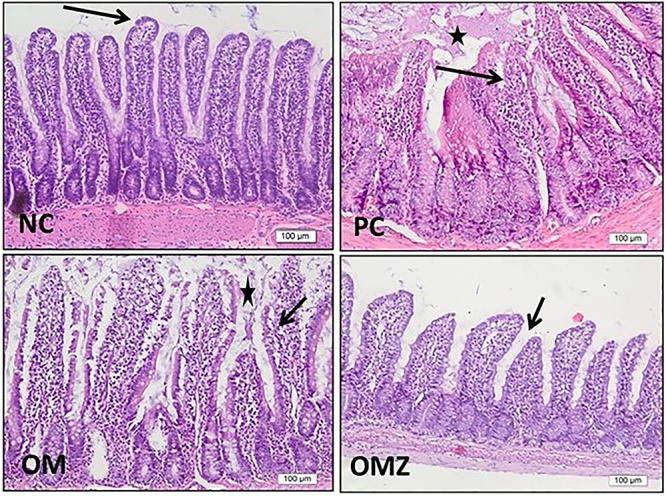
Photomicrographs of rat duodenal sections stained with H&E (×20, scale bar 100 μm). **NC**, The normal control group shows normal duodenal mucosa with normal villi, intact epithelium (arrow), and normal cellular core. **PC**, The positive control (indomethacin-induced duodenitis, untreated) group shows distorted hyalinized villi with damaged epithelium (arrow), luminal mucus (star), inflammatory cell infiltration, vascular congestion, and edema. **OM**, The olmesartan medoxomil group shows some intact normal villi (arrow) and highly swollen cellular core (star). **OMZ**, The olmesartan-medoxomil zeinmersomes group shows almost normal villi but with slight swelling and shortening, intact epithelium (arrow), and thin cores with normal cellularity.

The normal duodenum showed normal villus and crypt histoarchitecture, normal epithelial covering, and normal goblet cell population with an absence of luminal mucus (score 1). The duodenitis rats showed severe injury manifested by marked deformity and hyaline degeneration of both villi and crypts (villi were distorted, irregular, and swollen), damaged surface epithelium, and loss of goblet cells. The villi showed high density of inflammatory cell infiltration, vascular congestion, and luminal mucus (score 4). OM moderately improved these changes showing mild preservation of villus shape, surface epithelium, and goblet cells with moderate inflammatory cell infiltration and the presence of some luminal mucus (score 3). OMZ markedly improved these changes showing marked preservation of villus and crypt histoarchitecture, but with slight swelling and shortening. Goblet cells were nearly preserved with mild inflammatory cell infiltration and the presence of minimal luminal mucus (score 2) (Figure 2).

### Protein expression of survivin by western blot

**Figure 3:**
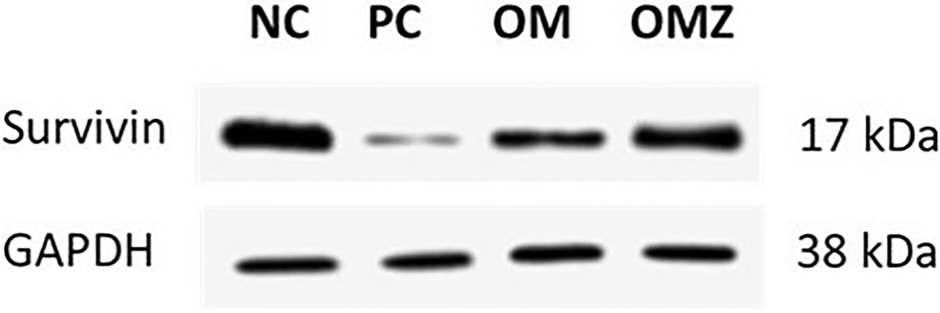
Western blot for protein expression of survivin (17 kDa). GAPDH (38 kDa) was used as an internal control in indomethacin-induced duodenitis in rats. The membrane was cut at molecular weight 30 kDa. Olmesartan medoxomil-zeinmersomes (OMZ) upregulated survivin expression more effectively than olmesartan medoxomil (OM) with a blot similar to that of the normal control (NC) group, while OM exerted just a moderate increase compared with the positive control (PC, indomethacin-induced duodenitis, untreated) group.

The indomethacin-induced duodenitis rats showed a decreased expression of survivin. OMZ more effectively upregulated survivin expression, compared with OM, with a blot similar to that of the normal control (Figure 3).

### Concentration of olmesartan in plasma and duodenal homogenate

**Figure 4:**
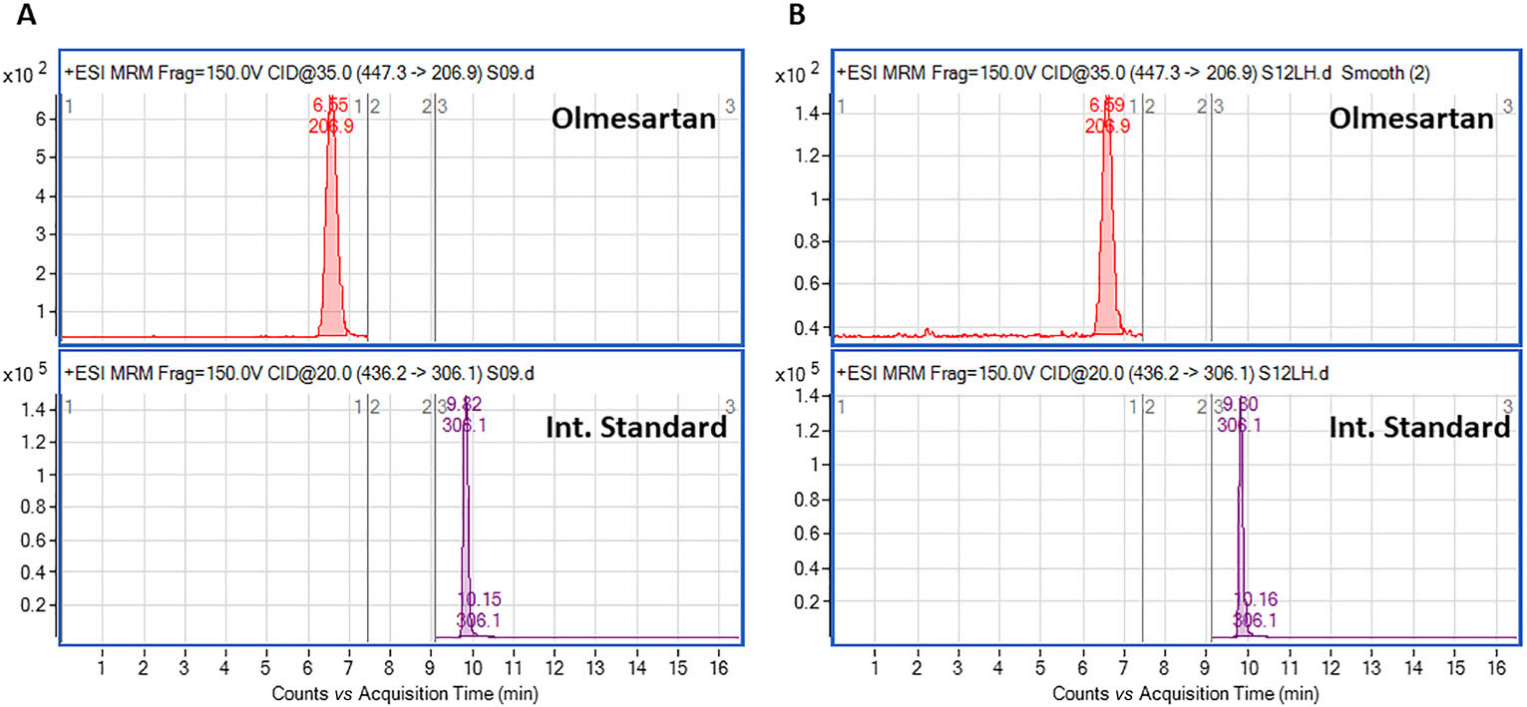
Chromatograms of olmesartan in (**A**) plasma and (**B**) duodenal homogenate.

The OMZ group achieved a significantly higher concentration of duodenal olmesartan than the OM group (duodenal/plasma ratio 6.07±1.02 *vs* 3.51±0.50, respectively). Figure 4 shows representative multiple reaction monitoring (MRM) transition chromatograms of olmesartan in plasma and duodenal homogenate.

## Discussion

Indomethacin is widely used to induce models of GI inflammation and ulceration possibly through inhibiting the defensive mechanisms and increasing the aggressive factors. Peptic ulcer disease is a common GI disorder and it has various causes, but *H. pylori* infection and NSAID use are the major causes (22). The duodenum is an overlooked segment in the literature and most studies, for example in GI radiology, focus on the stomach, distal small intestine, and colon (3). Although duodenitis can cause serious complications like GI bleeding, severe pain, and rapid weight loss, it usually improves with treatment (23). Based on the above-mentioned points, it is worthwhile to search for an effective and relatively safe treatment for duodenitis.

In the current study, the recently developed OMZ was more effective in protecting the duodenum against indomethacin-induced damage compared with OM, possibly due to improved bioavailability and better anti-inflammatory, antioxidant, and antiapoptotic effects. The current results agree with previously reported results in a model of duodenal ulceration induced by treating fasting rats with indomethacin and histamine dihydrochloride. Duodenal myeloperoxidase activity was significantly elevated, and antacids and prostaglandins protection against inflammation was significantly improved (24). Moreover, OM pretreatment improved indomethacin-induced gastric ulcer in rats where it decreased IL-6, TNF-α, and oxidative markers and improved mucosal contents of cyclooxygenase-1 and prostaglandin E2. Interestingly, the more bioavailable OM niosomes formula achieved superior gastro-protective effects compared to the standard OM form (25).

The NF-κB acts as an important link between oxidative and inflammatory pathways. It regulates expression of genes of the inflammatory pathway like TNF-α and IL-6, increasing inflammation and extension of ulcer injury. Thus, suppression of the NF-κB signaling pathway protects against ulcer development (26). In the current study, OMZ more effectively downregulated the indomethacin-induced increased gene expression of *NF-κB p65* in the duodenal mucosa. Previously, in acetic acid-induced ulcerative colitis in rats, oral OM inhibited NF-kB signaling and apoptosis. It decreased levels of colonic IL-6, TNF-α, IL-1β, transforming growth factor beta (TGF-β), neutrophil accumulation, *Bax*, and caspase-3 expression while it enhanced colon antioxidant mechanisms and the *Bcl-2*/*Bax* ratio (12). In acetic acid-induced duodenal ulcers, the Bax/*Bcl-2*/caspase-3 signaling becomes active and it is reported that drinking deep sea water improved ulcer through inhibition of this apoptotic signaling and upregulation of *Bcl-2* (27).

In the current study, western blot analysis for protein expression of survivin showed a decreased expression level in the indomethacin-induced duodenitis rats. OMZ upregulated survivin expression more effectively than OM with a blot similar to that of the normal rats, while OM exerted just a moderate increase compared with the disease model. Previously, it was reported that angiotensin II (Ang II) could be proapoptotic or antiapoptotic depending on the pathophysiological status of the cells. For example, the growth and migration of microvascular endothelial cells (EC) occurs during the initial phase of Ang II-mediated angiogenesis, while the following maturation and pruning of the neovasculature may involve EC differentiation and apoptosis (28). Ang II exerts its antiapoptotic effect through its interaction with its AT1 receptor, which activates the phosphatidylinositol-3-kinase/Akt (PI3K/Akt) pathway, stimulating the expression of the anti-apoptotic protein survivin (29). Angiotensin II could stimulate duodenal mucosal alkaline secretion by activating the AT2 receptors in the duodenal mucosa (30). In diabetic rats, an erythropoietin-derived peptide protected the heart against ischemia/reperfusion injury by stimulating anti-apoptotic mechanisms including up-regulation of myocardial survivin expression, mainly via PI3K/Akt mechanism (31).

It is well-known that the integrity of the gastric and duodenal mucosa depends on the balance between aggressive factors and protective mechanisms. The endothelium protects the mucosa through production of vasodilators (such as nitric oxide and prostaglandin I2) and release of angiogenic growth factors, ensuring appropriate blood supply. This microcirculation is vital for providing oxygen, nitric oxide, and hydrogen sulfide and eliminating toxic materials in addition to constant mucosal cell renewal from the progenitor cells ensured by growth factors and survivin preventing early apoptosis (32). Adequate mucosal blood flow of the GI greatly protects the mucosa and helps its regeneration, while a drop in blood supply increases mucosal sensitivity to damaging factors and promotes the development of ulcers. Induction of GI inflammation was associated with stimulation of the renin-angiotensin system, and suppression of the renin-angiotensin system enhanced blood flow, protecting GI tissues and accelerating regeneration (33). The oxidative stress induces expression of several genes in charge of apoptosis. OM downregulated caspase-3 and upregulated survivin expression, promoting the reduction of apoptosis. The decrease of duodenal apoptosis can be ascribed to the inhibition of oxidative stress because severe exposure of intestinal mucosa to reactive species in inflammatory conditions enhances epithelial apoptosis (34).

In a model of methotrexate-induced intestinal mucositis in rats, OM pretreatment decreased the inflammatory infiltration, ulceration, vascular congestion, and levels of myeloperoxidase, IL-1β, and TNF-α (13). The α2 adrenergic receptors may also be involved in GI inflammation, where their blocking increases the aggressive factors while their stimulation increases the protective factors (35). There is a cross talk between OM and α_1A_ adrenergic receptors: angiotensin II selectively downregulates them in rat cardiac myocytes (36) and OM suppresses cardiac α1 adrenergic receptor density (37).

In the current study, indomethacin decreased mucus production due to a marked loss of goblet cells, while OM and OMZ caused mild and moderate preservation of goblet cells, respectively, and thus both enhanced mucus production. This finding agrees with a previous study that reported that indomethacin decreased the small intestinal mucus content while lafutidine and capsaicin significantly increased it as a part of their protective action against indomethacin-induced intestinal ulceration in rats (38). For an accurate evaluation of the mucous content, periodic acid-Schiff (PAS) staining is required, and its absence was considered a limitation of the current study. In addition, for preparation of OMZ, small doses of zein and PEG were used, as explained in the methods section. Both zein and PEG have been proposed as topical protectors of the gastrointestinal tract mucosa. However, their potential protective effects occur in high concentrations, for example 5% PEG in drinking water protected the intestinal epithelium from microbial invasion in mice (39), and zein concentrations in zein-based gels ranged from 10 to 20% (40). The lack of an experimental group with the preparation of the nanoformulation without olmesartan to detect any protective effect of zein and PEG, if any, is considered another limitation of the current study.

In conclusion, the OMZ nano-formula achieved a higher duodenal drug concentration and a better improvement of the indomethacin-induced duodenitis compared with the regular OM in rats. It showed significantly better improvement of duodenal inflammation, oxidant stress, and histopathological alterations. Moreover, it downregulated gene expressions of *NF-κB p65* and *Bax*, upregulated gene expression of *Bcl-2*, increased the *Bcl-2*/*Bax* ratio, and increased protein expression of survivin, an important antiapoptotic protein. OMZ could be a better choice for hypertensive patients with NSAIDs-induced duodenitis. To our knowledge, this is the first study that reports such molecular mechanisms that explain the effects of a novel nano-formula of OM in a model of duodenitis similar to NSAID-induced duodenitis in humans.
